# Pectoral nerve II block, transversus thoracic muscle plane block, and dexmedetomidine for breast surgery in a patient with achondroplasia: a case report

**DOI:** 10.1186/s40981-019-0267-5

**Published:** 2019-07-19

**Authors:** Toshiyuki Nakanishi, Manabu Yoshimura, Takashi Toriumi

**Affiliations:** 1 Department of Anesthesiology, Japan Community Healthcare Organization Tokuyama Central Hospital, 1-1, Koda-cho, Shunan, Yamaguchi, 745-8522 Japan; 2 Present address: Department of Anesthesiology and Intensive Care Medicine, Nagoya City University Graduate School of Medical Sciences, Kawasumi 1, Mizuho-cho, Mizuho-ku, Nagoya, 467-8601 Japan

**Keywords:** Achondroplasia, Pectoral nerve block, Transversus thoracic muscle plane block

## Abstract

**Background:**

Patients with achondroplasia have various airway deformations and spinal anatomic abnormalities; therefore, performing general anesthesia and neuraxial anesthesia in such patients can be challenging.

**Case presentation:**

A 56-year-old, 112-cm, 30-kg woman was scheduled to undergo partial mastectomy and sentinel lymph node biopsy for cancer of the right breast. She had short limbs, scoliosis, thorax deformation, and chronic moderate to severe mitral regurgitation of the mitral valve. We performed pectoral nerve II block and transversus thoracic muscle plane block and administered intravenous dexmedetomidine. The surgery was completed without the administration of any additional analgesics or sedatives.

**Conclusions:**

We successfully performed breast surgery using pectoral nerve II block, transversus thoracic muscle plane block, and sedation with dexmedetomidine in a patient with achondroplasia. We found that the combination of peripheral nerve blocks is a useful option in patients who have difficulties with both general anesthesia and neuraxial anesthesia.

## Background

Achondroplasia is the most common form of short-limb dwarfism, occurring in approximately 1 in 20,000 live births [1]. Patients with achondroplasia have various anatomic deformations, such as midface hypoplasia, atlantoaxial instability, and enlarged oropharyngeal soft tissues, which consequently result in obstructive sleep apnea, restrictive lung disease, and, possibly, difficult airways [2]. Patients with achondroplasia also have spinal anatomic abnormalities, such as spinal stenosis, scoliosis, and malformed vertebral bodies [3]. Because of these abnormalities, general anesthesia and neuraxial anesthesia can be challenging in these patients.

Ultrasound-guided peripheral nerve block has been developed for pain relief in various types of surgery. Blanco et al. have described pectoral nerve I (Pecs I) block, targeting an interfascial plane between the pectoralis major and pectoralis minor muscles, aiming to block the lateral region of the breast [4]. Subsequently, Pecs II block, which comprises Pecs I block as the first injection and a second injection at the interfascial plane between the pectoralis minor muscle and serratus anterior muscle for analgesia of the axilla, was reported to be an effective analgesia for breast surgery [5, 6]. Recently, Ueshima et al. have reported transversus thoracic muscle plane (TTP) block for analgesia of the inner breast region [7]. With the combination of these techniques, breast surgery using superficial peripheral nerve blocks can be performed in patients who have difficulties with both general anesthesia and neuraxial anesthesia. Here, we report a case of a patient with achondroplasia who underwent breast surgery using Pecs II block, TTP block, and sedation with dexmedetomidine.

## Case presentation

A 56-year-old, 112-cm, 30-kg woman was scheduled to undergo partial mastectomy in the right upper inner to the outer region of the breast and sentinel lymph node biopsy for cancer of the right breast. She had short limbs, scoliosis, and thorax deformation because of achondroplasia, but she used a wheelchair and maintained activities of daily living. She had received carvedilol, losartan, and furosemide for chronic moderate to severe mitral regurgitation due to shortening of the posterior leaflet of the mitral valve. She had a history of catheter ablation for paroxysmal atrial tachycardia; however, her preoperative electrocardiogram revealed normal sinus rhythm. She showed no signs of respiratory symptoms with exercise; however, she could not maintain a supine position for a long period because of shortness of breath. A preoperative respiratory function test revealed a volume capacity of 1.51 L and a forced expiratory volume in 1 second of 1.21 L. Preoperative examination revealed normal opening of the mouth, a Mallampati grade of 2, and normal flexion of the neck. We weighed the risk of general anesthesia for respiratory and cardiac functions of the patient and possible difficult airway against the feasibility of intraoperative management without general anesthesia. Consequently, intraoperative management using Pecs II block, TTP block, and sedation with dexmedetomidine was planned.

**Fig. 1:**
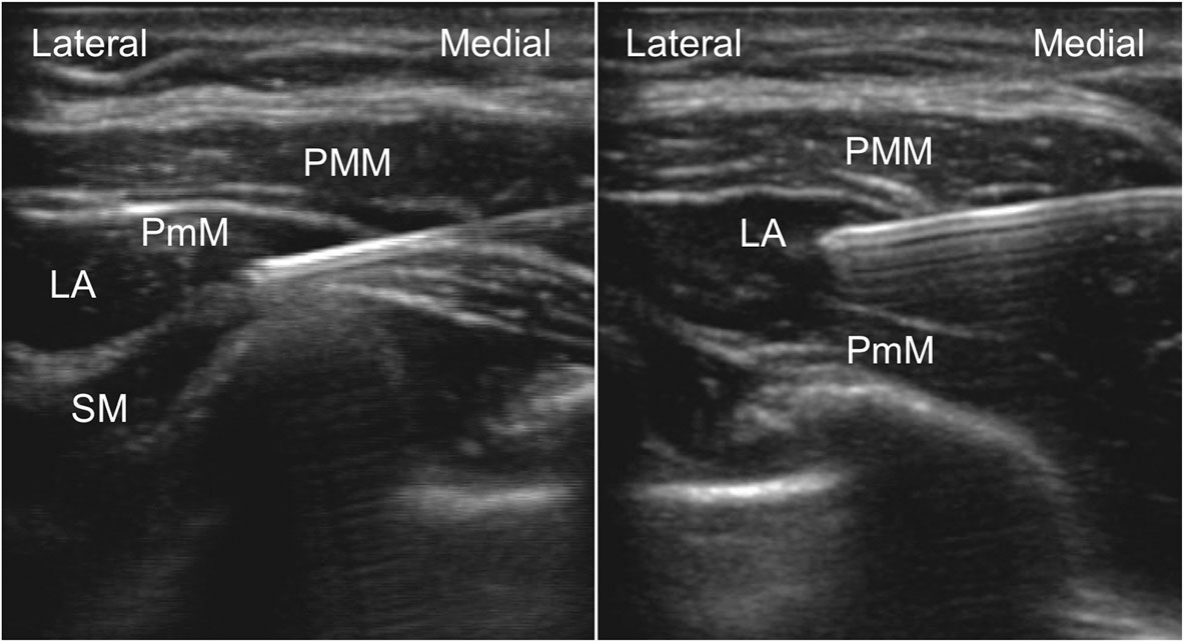
Ultrasound images of pectoral nerve II block. The left image shows injection of local anesthetics between the pectoralis minor muscle and the serratus anterior muscle. The right image shows injection of local anesthetics between the pectoralis major muscle and the pectoralis minor muscle. LA, local anesthetics; PmM, pectoralis minor muscle; PMM, pectoralis major muscle; SM, serratus anterior muscle

**Fig. 2:**
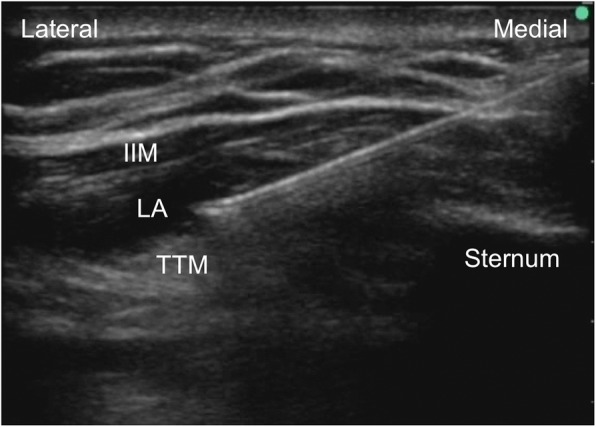
Ultrasound image of transversus thoracic muscle plane block. Local anesthetics were spread between the internal intercostal muscle and the transversus thoracic muscle. IIM, internal intercostal muscle; LA, local anesthetics; TTM, transversus thoracic muscle

In the operating room, the patient was monitored for non-invasive blood pressure, electrocardiogram, and peripheral oxygen saturation. After the commencement of oxygen inhalation at 2 L/min via a nasal cannula, intravenous dexmedetomidine at 6 μg/kg/h was administered for 10 min and thereafter at 0.4 μg/kg/h. After infiltration of each puncture site with 5 mL of 1% lidocaine, the following ultrasound-guided blocks were performed with a 70-mm long 22-gauge Tuohy needle and ultrasound equipment SonoSite Edge (FUJIFILM SonoSite, Bothell, WA, USA). Firstly, Pecs II block was performed. The operator stood at the head side of the patient and placed the ultrasound probe at the caudal side of the clavicle. After identifying the plane between the pectoralis minor muscle and serratus anterior muscle at the fourth rib, the needle tip was positioned in this plane and 15 mL of 0.25% levobupivacaine was injected. The needle tip was then pulled back to the plane between the pectoralis major and pectoralis minor muscles, and 10 mL of 0.25% levobupivacaine was injected (Fig. 1). Secondly, TTP block was performed. The operator stood at the left side of the patient and placed the ultrasound probe at the right lateral border of the sternum between the third and fourth ribs. The needle was advanced to the plane between the internal intercostal muscle and transversus thoracic muscle with an in-plane approach, and 11 mL of 0.25% levobupivacaine was injected (Fig. 2). The longitudinal spread of local anesthetics between these muscles from the second to the fifth rib was ultrasonographically confirmed immediately after the injection. Sensory blockade of the inner to outer region of the right breast at the Th2–5 of the intercostal nerve and right axilla was confirmed by a pin-prick test performed 15 min after the blocks, and the surgery was initiated. During surgery, the administration of additional analgesics or sedatives was not required because the patient did not complain of pain. She complained of dyspnea twice during surgery, but her symptoms improved after she was encouraged to take several deep breaths and the dexmedetomidine dosage was increased to 0.7 μg/kg/h. During surgery, her hemodynamic state was stable, peripheral oxygen saturation was maintained at 99–100%, and respiratory rate was 18–22/min. As there was no metastasis in the sentinel lymph node, the surgery was completed after partial mastectomy and sentinel lymph node biopsy, with a total operation time of 37 min. Before completion of the surgery, 30 mg of flurbiprofen and 450 mg of acetaminophen were intravenously administered for postoperative multimodal analgesia. Immediately after the surgery, the patient rated her pain as 0/10 on the numeric rating scale. The next morning after the surgery, she rated her pain as 0/10 at rest and 1/10 while moving, without additional analgesics. The patient had a good postoperative course and was discharged from the hospital 2 days after the surgery.

## Discussion

We performed successful management of breast surgery using Pecs II block, TTP block, and sedation with dexmedetomidine in a patient with achondroplasia. This shows that the combination of Pecs II and TTP blocks with intravenous dexmedetomidine can be an alternative to general anesthesia and neuraxial anesthesia in patients who have difficulties with them.

Patients with achondroplasia have potential problems for both general anesthesia and neuraxial anesthesia. For general anesthesia, difficult airway associated with obstructive sleep apnea, atlantoaxial instability, and adenotonsillar hypertrophy can be a problem. Some investigators have reported difficult airway in patients with achondroplasia [8, 9]. The strategy of airway management should be planned for individual patients because achondroplasia causes deformities in various parts of the airway and its severity may vary widely according to patients. We planned to avoid the use of general anesthesia, considering the feasibility of management with peripheral nerve blocks and sedation and the risk of general anesthesia for respiratory and cardiac functions and potential airway difficulties in the patient. Epidural anesthesia or thoracic paravertebral block can be used for pain relief in breast surgery [10, 11]. However, spinal abnormalities in patients with achondroplasia can make neuraxial anesthesia or deep peripheral nerve block difficult. Therefore, we decided to use the combination of truncal regional anesthesia and intravenous dexmedetomidine. Pecs I block targets the lateral and medial pectoral nerves and blocks the lateral mammary area [4], whereas Pecs II block targets the intercostobrachial, long thoracic, and multiple upper intercostal nerves and produces sensory loss in the axillary region in addition to the area affected by Pecs I block [5]. Although Pecs I and Pecs II blocks cannot block the inner region of the breast, TTP block targets multiple anterior branches of the intercostal nerves and blocks the internal mammary area [7]. These peripheral nerve blocks have the following advantages: they can target relatively superficial regions and there is no need to change the patient’s position. Because of these advantages, the use of superficial peripheral nerve blocks, such as Pecs II or TTP blocks, may be prudent for patients with achondroplasia as shown in this report.

In breast surgery, epidural anesthesia or thoracic paravertebral block without general anesthesia have been reported as good analgesia [11]. In contrast, reports on the combination of Pecs II and TTP blocks without general anesthesia are limited. Ueshima et al. have reported Pecs II and TTP blocks without general anesthesia for the segmental upper outer region of the left breast in an 86-year-old patient with low cardiac function [7]. Thereafter, they reported bilateral Pecs II and TTP blocks without general anesthesia for bilateral breast resection in a patient with severe obstructive pulmonary disease [12]. Kim et al. have reported Pecs II block and internal intercostal plane block without general anesthesia for a huge breast fibroadenoma resection in a patient with fear for general anesthesia [13]. Hong et al. have reported modified Pecs II block (below the serratus muscle as the second injection) and pecto-intercostal fascial block without general anesthesia for partial resection of recurrent breast cancer in a parturient [14]. Compared with these reports, our case was challenging in terms of determining the dose of local anesthetics because the body weight of the patient was only 30 kg. According to these previous reports, Pecs II block requires 10 and 20 mL of local anesthetics for the shallower and deeper plane, respectively, and TTP block requires 10–15 mL of local anesthetics. Therefore, we considered to use a total of 40–45 mL of local anesthetics. As the body weight of our patient was 30 kg, we could administer up to 36 or 45 mL of 0.25% levobupivacaine or 0.2% levobupivacaine, respectively, considering the maximum dose of 3 mg/kg. A total of 36 mL of 0.25% levobupivacaine was considered because we believed that a lower concentration would be inadequate without general anesthesia management. Thus, the dose of local anesthetics was reduced for the lower region of the breast. Sensory loss around the incisional area at the right upper breast and axilla region was confirmed, and intraoperative management was successfully performed without any additional analgesics.

In summary, we report the successful management of breast surgery in a patient with achondroplasia using Pecs II block, TTP block, and sedation with dexmedetomidine. The combination of peripheral nerve blocks is a useful option in patients who have difficulties with both general anesthesia and neuraxial anesthesia.

## Data Availability

Not applicable.

## Abbreviations

Pecs: Pectoral nerve
TTP: Transversus thoracic muscle plane
